# Estimation of Soil Arsenic Content with Hyperspectral Remote Sensing

**DOI:** 10.3390/s20144056

**Published:** 2020-07-21

**Authors:** Lifei Wei, Haochen Pu, Zhengxiang Wang, Ziran Yuan, Xinru Yan, Liqin Cao

**Affiliations:** 1 Faculty of Resources and Environmental Science, Hubei University, Wuhan 430062, China; 201722110813023@stu.hubu.edu.cn (H.P.); wangzx66@hubu.edu.cn (Z.W.); 201711110811066@stu.hubu.edu.cn (Z.Y.); 201811110811201@stu.hubu.edu.cn (X.Y.); 2 Hubei Key Laboratory of Regional Development and Environmental Response, Hubei University, Wuhan 430062, China; 3 State Key Laboratory of Information Engineering in Surveying, Mapping and Remote Sensing, Wuhan University, Wuhan 430079, China; 4 School of Printing and Packaging, Wuhan University, Wuhan 430079, China; clq@whu.edu.cn

**Keywords:** hyperspectral remote sensing, arsenic, sCARS-SPA, SFLA-RBFNN

## Abstract

With the continuous application of arsenic-containing chemicals, arsenic pollution in soil has become a serious problem worldwide. The detection of arsenic pollution in soil is of great significance to the protection and restoration of soil. Hyperspectral remote sensing is able to effectively monitor heavy metal pollution in soil. However, due to the possible complex nonlinear relationship between soil arsenic (As) content and the spectrum and data redundancy, an estimation model with high efficiency and accuracy is urgently needed. In response to this situation, 62 samples and 27 samples were collected in Daye and Honghu, Hubei Province, respectively. Spectral measurement and physical and chemical analysis were performed in the laboratory to obtain the As content and spectral reflectance. After the continuum removal (CR) was performed, the stable competitive adaptive reweighting sampling algorithm coupled the successive projections algorithm (sCARS-SPA) was used for characteristic band selection, which effectively solves the problem of data redundancy and collinearity. Partial least squares regression (PLSR), radial basis function neural network (RBFNN), and shuffled frog leaping algorithm optimization of the RBFNN (SFLA-RBFNN) were established in the characteristic wavelengths to predict soil As content. These results show that the sCARS-SPA-SFLA-RBFNN model has the best universality and high prediction accuracy in different land-use types, which is a scientific and effective method for estimating the soil As content.

## 1. Introduction

The pollution of arsenic (As) to the environment has caused widespread concern all over the world [1], and harm to the body of local residents has been confirmed in recent years. Once it enters the human body, no matter through what way, only a small amount will be eliminated, most will accumulate in the body. Arsenic accumulated in the human body will cause abnormal cell metabolism after destroying the redox ability of cells, resulting in serious diseases and even death. It is recognized as a top pollutant by the US Toxic Commission [2]. In arsenic-contaminated areas, the toxicity and biological activity of arsenic depend on the form of arsenic. Among all forms, As (V) accounts for the vast majority, and has the highest biological effectiveness [3]. Therefore, the determination of total arsenic in the soil can reflect the pollution of arsenic in most areas, which is of great significance.

Considering the importance of soil safety to human health and agricultural development, it is very important to identify areas contaminated by heavy metals in the soil [4]. For this reason, a low-cost and feasible technique for estimating soil heavy metal content is required in environmental monitoring. Conventionally, multiple soil samples and chemical analysis is necessary for the estimation of heavy metal content in soil, which is expensive and inefficient. At the same time, on account of the certain spatial heterogeneity of arsenic distribution in soil [5], laboratory measurement results are difficult to be applied quickly to a wide range. After hyperspectral remote sensing was widely used in vegetation science and crop management, it has provided the possibility of large-scale, low-cost, and rapid detection of pollutants in soil [6].

The use of hyperspectral data is divided into the use of the full band and the selection of characteristic wavelengths. When using the full band, the problem of data redundancy is obvious due to the high spectral resolution. For the purpose of simplifying the model and improving the prediction accuracy, the characteristic wavelength of hyperspectral data is selected [7]. The model established in the characteristic wavelengths selected by the competitive adaptive reweighting sampling algorithm (CARS) coupled with other methods also has excellent performance. Xu et al. [8] analyzed soil Zn pollution in the Weibei Plain in northern Shandong Province. The results show that the CARS method has a good performance in selecting feature bands which are compared and evaluated with the full-band based on the performance of models. Tan et al. [9] obtained the characteristic wavelengths based on CARS coupled with partial least squares (CARS-PLS) and found that CARS-PLS is a promising method to eliminate redundant bands, and the prediction accuracy of the model established by the selected characteristic wavelength is higher than CARS. Yuan et al. [10] applied Spearman’s rank correlation analysis coupled with CARS (CARS-SCA) for the prediction of soil As content, and the results showed that CARS-SCA could make the model simpler and more accurate than CARS. As an improvement to the CARS method, stable CARS (sCARS), which uses variable stability as a measurement indicator, has proven to be an excellent method for selecting feature variables [11,12]. The successive projections algorithm (SPA) can solve the problem of collinearity between spectral variables effectively [13], but the sCARS coupled with SPA is rarely used to select characteristic wavelengths in the field of estimating the soil As content.

In the analysis of hyperspectral data, there are many modeling methods. Among modeling methods, partial least squares regression (PLSR) is the most common regression algorithm for hyperspectral data [14]. In addition to statistical analysis models like PLSR, in recent years, the neural network model also has good performance for estimating the soil heavy metal content. Zhao et al. [15] took the soil sample of Guangdong Province as an example and used the genetic algorithm optimization of the back-propagation neural network (GA-BPNN) model to establish a hyperspectral heavy metal mercury content prediction model. With the characteristics of fast convergence and global approximation, the radial basis function neural network (RBFNN) is more convenient and accurate than the BPNN [16], and is an excellent fitting method. Zhang et al. took the mining wasteland as the research object, and estimated the heavy metal content for reclaimed soil samples based on RBFNN [17]. As a famous metaheuristic optimization technique, the shuffled frog leaping algorithm is an efficient global optimizer. However, few studies have used a combination of these two methods to study the prediction of soil As content. Therefore, in this paper, based on the characteristic wavelength selected by sCARS-SPA, shuffled frog leaping algorithm (SFLA) is used to optimize the RBFNN algorithm, and applied to estimating the As content in soil.

In this paper, the research objects are the soil samples in Honghu and Daye, Hubei Province, China. The purpose is to compare the accuracy of the soil As content estimation model established by different characteristic wavelength selection methods and explore a more suitable estimation model. The specific goals are: (1) explore the characteristic wavelengths in the prediction of soil As content; (2) explore the ability of continuum removal (CR) to enhance the effect of characteristic wavelength selection method; (3) characteristic wavelengths were selected by sCARS and sCARS-SPA, separately, and the effects of the two characteristic wavelength selection methods were compared on different models; (4) compare the modeling results of PLSR, RBFNN, and SFLA-RBFNN to find a method of soil As content with high robustness and prediction accuracy.

## 2. Materials and Methods

### 2.1. Study Area

**Figure 1:**
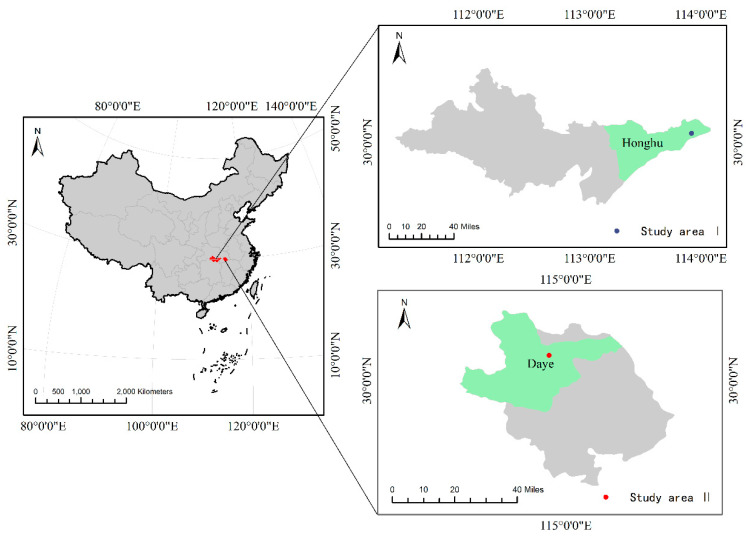
Study area locations.

The study area is located in Honghu and Daye, Hubei Province, China. Daye (114°31′~115°20′ E, 29°40′~30°15′ N) has a total area of 1566.3 km^2^, of which cultivated land area is about 480.1 km^2^. In a subtropical continental monsoon climate, Daye has sufficient sunlight and precipitation, which is conducive for agricultural production. Daye has rich mineral resources [18] and more than 2000 years of mining and smelting history. The mining and smelting, which is mainly based on copper and iron ore mining, have promoted the rapid economic development of the region. At the same time, the excessive exploitation of resources has also caused serious pollution of the region’s soil, posing a threat to the development of the agricultural economy. In Daye, the land-use type of the soil sample collection area belongs to farmland. Honghu (113°07′~114°05′E,29°39′~30°12′N) has a total area of 2519 km^2^. In a subtropical humid monsoon climate, winter and summer in Honghu are longer than spring and autumn, and the precipitation is also abundant. In recent years, due to industrial production and transportation, the soil heavy metal pollution is more serious than before, which has led to the deterioration of the ecological environment quality. The land-use type of the soil sample collection area belongs to industrial and mining land. The location of the study area is shown in Figure 1.

### 2.2. Research Methods

#### 2.2.1. The sCARS-SPA Characteristic Wavelength Selection Algorithm

Founded on Monte Carlo sampling (MCS) and partial least squares regression coefficients, CARS is considered to be a popular and effective characteristic wavelength selection algorithm [19]. However, the variable regression coefficient will change with each random selection of modeling samples, and the importance of the wavelength cannot be well reflected by the absolute value of the regression coefficient. Zheng et al. [12] proposed the sCARS algorithm, which takes the stability of variables as the indicator to measure the importance of variables, and continues the variable screening process of CARS. In addition, there is collinearity among spectral characteristic wavelengths. SPA can select the wavelength group containing the most relevant information to eliminate collinearity in wavelengths as much as possible [20]. In this study, SPA was used to perform second characteristic wavelength selection to eliminate the collinear effect among many wavelength variables. The specific process of the sCARS-SPA is as follows:

Step 1: Calculate the stability index of each wavelength

Based on the stability index, the importance of wavelengths is directly reflected. The spectral data $X_{p\times q}$ contains q spectral responses of p samples.$\;y_{q\times 1}$ is the arsenic content of q samples. The formula about $X_{p\times q}$ and $y_{q\times 1}$ can be expressed as:

(1) K=Xα+F

where α is a regression coefficient vector, the number of coefficients is q, and F is an error vector.

In the process of MCS, $q_{s}$ ($q_{s}<q$) samples are randomly selected from q samples, and the regression coefficient α is calculated. After R times of sampling, a matrix$\;A({\left[\alpha_{1},\alpha_{2},\ldots \alpha_{R}\right]}^{t}$ will finally be determined, which contains R corresponding regression coefficient vectors. The number of rows and columns of the R matrix are q and R, respectively. Stability is defined here as [21,22]:

(2) $$e_{i}=\left|\frac{{\;\bar{d}}_{i}}{s\left(d_{i}\right)}\right|$$

where for the ith wavelength, $e_{i}$, ${\bar{d}}_{i},$ and $s\left(d_{i}\right)$ are the stability index, the mean value, and the standard deviation, respectively, after R sampling runs. To ensure that the stability is positive when compared, the symbol of absolute value is applied to the formula.

Step 2: Select the group of wavelengths based on sCARS

The important wavelengths were obtained by mandatory wavelength selection and adaptive reweighted sampling (ARS) depending on the wavelength stability. For obtaining the best subset of variables at this stage, cross-validation is performed and the wavelength group with the minimum root mean square error of cross-validation (RMSECV) is the best wavelength group selected by sCARS.

Step 3: SPA is used to perform second feature selection on the variable subset obtained in step 2, eliminating the collinearity effect among many wavelength variables.

Specific steps are as follows:

(a) Initialization: z=1 (first iteration). In the wavelength group obtained in step 2, a wavelength $x_{j}$ is chosen by random selection.$\;x_{j}$ is expressed as $x_{r\left(0\right)}$, that is, $x_{r\left(0\right)}=\;j$;
(b) The set C is defined as:
  (3) C ={ j,1 ≤ j ≤ R, j∉{ r(0)⋯r(z −1) }}
  where R is the number of wavelengths. That is, the wavelength selected in the initialization has not been selected into the wavelength chain. The projection vector of $x_{j}$ to the vector in C is calculated.
  (4) $${\mathrm{Px}}_{j}=x_{j}-\left(x_{j}^{T}x_{r\left(z-1\right)}\right)x_{r\left(z-1\right)}{\left(x_{j}^{T}x_{r\left(z-1\right)}\right)}^{-1}$$
(c) The sequence number of the maximum projection is recorded
  (5) $$r\left(z\right)=\arg\left(\max\|{\mathrm{Px}}_{j}\|,j\in C\right)$$
(d) The projection vector of the next round is the maximum projection of the previous round
  (6) $$x_{j}={\mathrm{Px}}_{j},\;j\in C$$
(e) z=z+1, if z<R, go back to (b) to continue projection.

For each pair of $x_{r\left(0\right)}$ and R, the RMSECV of the calibration set is obtained on multiple linear regression analysis (MLR). The $x_{r\left(0\right)}$ and R corresponding to the smallest RMSECV are the final subsets of wavelengths.

#### 2.2.2. Partial Least Squares Regression

As a commonly used multivariate statistical algorithm, PLSR considers not only the extraction of principal components from dependent and independent variables, but also the maximization of the correlation between principal components extracted from independent and dependent variables [23,24]. Therefore, PLSR is a general method of modeling using hyperspectral data.

#### 2.2.3. SFLA-RBFNN Method for Estimation of the Soil As Content

**Figure 2:**
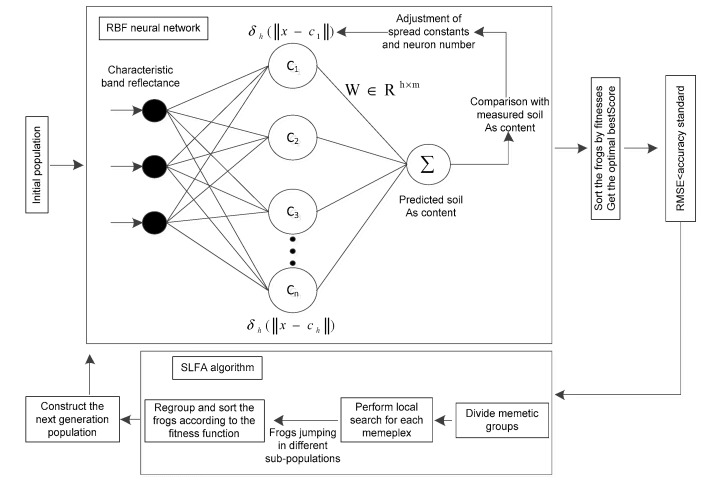
The structure of shuffled frog leaping algorithm optimization of the radial basis function neural network (SFLA-RBFNN).

RBFNN can handle the difficulty in analyzing regularity and approximate any non-linear function, and is considered to be excellent in generalization and convergence rate. As shown in Figure 2. RBFNN is a feedforward neural network with a three-layer structure, including input layer, hidden layer, and output layer. 

The nonlinear function $h\left(x,c_{i}\right)$ is used as a radial basis function at each node of the hidden layer. The commonly used radial basis transfer function is a Gaussian kernel function, and its formula is:

(7) $$h\left(x,c_{i}\right)=e^{-\frac{\|x-c_{i}\|{}^{2}}{r_{i}^{2}}},i=1,2,3...,n$$

where${\;r}_{i}$ is the spread constant of the radial basis function, $c_{i}$ is the value of the center symmetry point, and i is the number of neurons. After the radial basis function is determined, the output value of the RBF neural network can be obtained by linearly summing the function results.

SFLA is a heuristic group optimization algorithm [25]. Combining the advantages of memetic algorithm and particle swarm optimization algorithm, it has excellent global optimization capabilities and computational efficiency. Therefore, in this study, the parameter of the RBFNN will be optimized by SFLA for improving the prediction accuracy and the robustness. The structure of SFLA-RBFNN is shown in the Figure 2.

To avoid the appearance of overfitting in the optimization process, cross-validation is performed during the establishment of the model. The individual fitness function is set to the mean value of the root mean square error (RMSE) of the validation set. The optimization objective function is:

(8) $$\mathrm{minE}=\frac{\sum_{k=1}^{K}\sqrt{\frac{\sum_{i=1}^{k}{\left(y_{i,k}-{\hat{y}}_{i,k}\right)}^{2}}{n}}}{K}$$

where K is the fold of cross-validation (here it is 4)$,\;y_{i,k}$ is the measured value of the verification set in the kth cross-validation, ${\hat{y}}_{i,k}$ is the predicted value of validation set in the kth cross-validation, and n is the number of samples in the validation set.

#### 2.2.4. Flowchart

**Figure 3:**
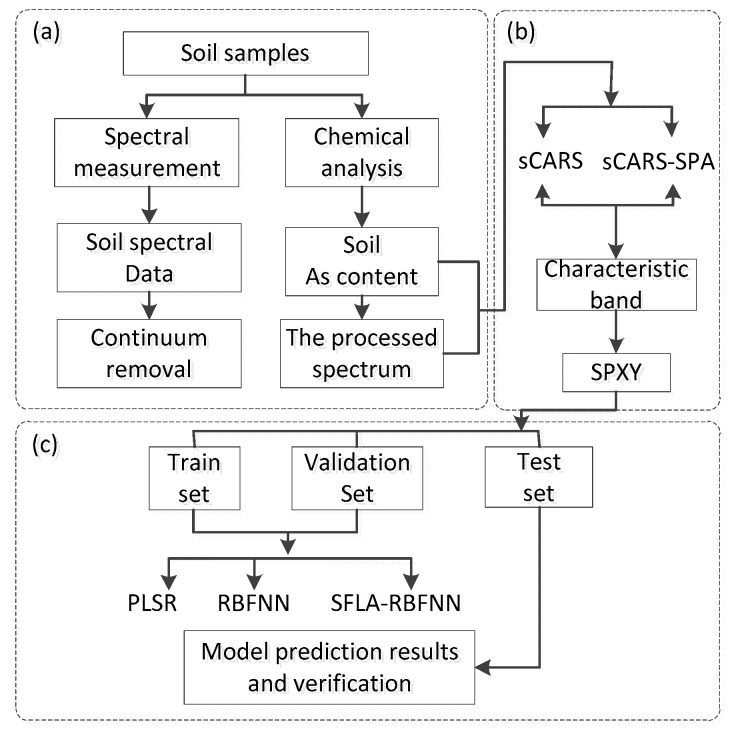
The flowchart for estimation of soil arsenic (As) content: (**a**) data acquisition and preprocessing; (**b**) characteristic wavelength selection; (**c**) modeling and verification.

The flowchart for prediction of soil As content is shown in Figure 3 and is mainly divided into the following four parts: (1) the acquisition and processing of soil hyperspectral data and the As content in soil; (2) the characteristic band is selected by sCARS and sCARS-SPA, respectively; (3) through the joint x−y distance (SPXY) algorithm, train set, validation set, and test set are obtained from the sample set; (4) PLSR, RBFNN, and SFLA-RBFNN are established in the characteristic wavelengths to predict soil heavy metal As content, and the model with the best accuracy is obtained.

### 2.3. Accuracy Evaluation

The performance of estimation models was evaluated by $R^{2}$, RMSE, and mean absolute error (MAE). The calculation formulae of $R^{2}$, RMSE, and MAE are as follows:

(9) $$R^{2}=1-\frac{\sum_{i=1}^{n}{\left({\;\hat{y}}_{i}-y_{i}\right)}^{2}}{\sum_{i-1}^{n}{\left(y_{i}-\;\bar{y}\right)}^{2}}$$

(10) $$\mathrm{RMSE}=\sqrt{\frac{\sum_{i=1}^{n}{\left(y_{i}-{\;\hat{y}}_{i}\right)}^{2}}{n}}$$

(11) $$\mathrm{MAE}=\frac{1}{n}\overset{n}{\underset{i=1}{\sum }}\left|y_{i}-{\;\hat{y}}_{i}\right|$$

where $y_{i}$ is the measured As content in the soil, ${\hat{y}}_{i}$ is the estimated soil As content, $\bar{y}$ is the mean of the measured soil As content, and n means the number of soil samples.

### 2.4. Experimental Procedure

#### 2.4.1. Soil Sample Collection and Processing

In Honghu and Daye, 27 and 62 soil samples were collected using a checkerboard sampling method at a depth of 0–20 cm. The soil samples were yellow-brown, rich in organic matter, and weakly acidic. According to the World References Based Soil Resources (IUSS WG WRB, 2015) [26], they belong to Fluvisols. After the collection was completed, each sample was split into two parts. The two parts were regarded as the targets of spectrum measurement and soil As content measurement, respectively. The measurement of As content was mainly based on laboratory analyses. First, impurities such as stones and plant roots in soil samples were removed as much as possible. After that, a sieve with a pore size of 2 mm was used to screen the crushed soil. Next, the sieved sample was compacted and screened again with a sieve with a pore size of 0.15 mm [27]. Finally, nitric acid, hydrochloric acid, and perchloric acid were used to decompose the arsenic in various forms in the soil sample, which converted them into soluble arsenic ions in the solution and avoided the interference of sulfur and phosphorus on the measurement. Then, potassium borohydride/silver nitrate spectrophotometry was used to measure the arsenic content based on the absorbance (it is assumed that the possible interference from other ions is acceptable) [3]. The determined value of each soil sample was calculated based on arithmetic average after three measurements.

#### 2.4.2. Soil Spectrum Collection and Processing

For the samples from Honghu, the spectral reflectance was measured by SVC HR-1024 field spectrometer. The number of wavelengths in the spectrum was 990. The resolutions of 350–1000 nm, 1000–1900 nm, and 1900–2500 nm were 1.5 nm, 3.8 nm, and 2.5 nm, respectively. The spectral reflectance of the soil sample from Daye was measured by ASD FieldSpec3 field spectrometer with spectral resolution of 1 nm and wavelength range of 350–2500 nm. The number of wavelengths is 2151. The soil sample was placed in a darkroom, where a 1000-watt halogen lamp was used as a light source. Perpendicular to the soil surface, the irradiation angle of the light probe was 45° [28].

**Figure 4:**
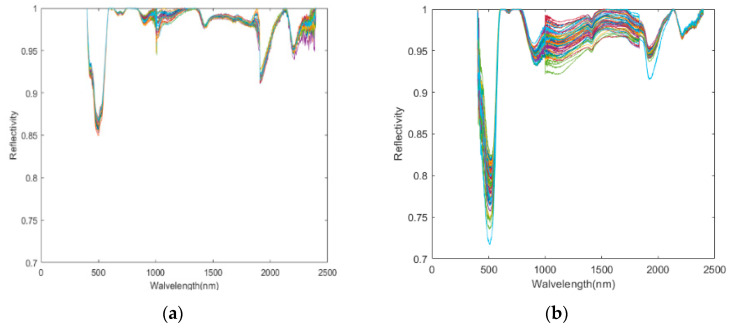
Spectral reflectance curve of soil sample after the removal of the noise edge wavelengths and the CR: (**a**) Honghu; (**b**) Daye.

During the measurement process of the spectrometer, although the spectrometer has a high measurement accuracy, there is usually a certain error between the measured content and the actual content due to the influence of factors such as the sample and the lighting conditions. Spectral transformation is a method to effectively emphasize the spectral characteristics and solve the problem of background noise. In this study, after performing the removal of the noise edge wavelengths of 350–399 nm and 2400–2500 nm [29], CR [30] was performed to highlight the absorption and reflection features of spectral curves, facilitating the extraction of feature wavelengths. The final results are used for modeling and analysis. After the processing is completed, the spectral reflectance curve of the soil sample from Honghu and Daye areas is shown in Figure 4.

### 2.5. Calibration Set, Validation Set, and Test Set

In order to consider both the soil As content vector and the spectral vector, the soil samples were split into a model set and test set for modeling and testing by SPXY algorithm [31], respectively. In the process of parameter optimization, for the purpose of avoiding the poor generalization of the model due to overfitting, the original training set is split into a calibration set and a verification set in the same proportion for cross-validation. Multiple groups of different training and validation of the model can also solve the problem of too one-sided and insufficient training data due to the results of individual testing. The validation set is used to evaluate the model for parameter optimization. The test set is used to evaluate the performance of the final model. For Daye, the number of training sets, verification sets, and test sets is 35, 12 and 15, respectively. For Honghu, the number of training sets, verification sets, and test sets is 15, 5 and 7, respectively. The algorithm selects the model with minimum generalization error as the final model and trains the model again on the whole training set to obtain the final model.

As shown in Table 1, according to the Soil Environmental Quality Standards GB15618-1995 in China, the mean of soil As content in the sampling area of Honghu is 33.05 ug/g, which exceeds the pollution standard. The mean of soil As content in the sampling area of Daye is 9.28 ug/g, which is lower than the pollution standard. Therefore, the sampling area of Honghu belongs to the contaminated area, and the sampling area of Daye belongs to the uncontaminated area. Two areas with different heavy metal As pollution levels are used as research areas, which can enhance the credibility and practicality of the experimental results.

## 3. Results

### 3.1. sCARS-SPA Characteristic Wavelength Selection Algorithm

**Figure 5:**
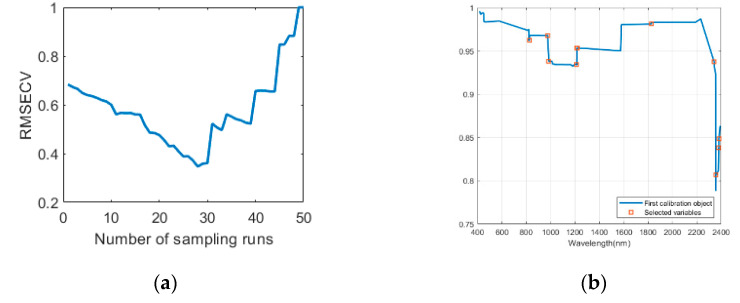
In Daye area, the stable competitive adaptive reweighting sampling algorithm coupled the successive projections algorithm (sCARS-SPA) is used for characteristic wavelength selection: (**a**) the root mean square error of cross-validation (RMSECV) of the 5-fold cross-validation changes with the increase of the number of sCARS algorithm sample runs; (**b**) the final characteristic wavelength and reflectance selected by SPA.

The sCARS-SPA algorithm can gradually eliminate redundant variables and collinear variables in soil hyperspectral data. The number of MCS samples is set to 50 and the fold of cross-validation is five. For the Daye area, after the five-fold cross-validation, the RMSECV trends of the CR of the reflectance spectra are shown in Figure 5. As shown in Figure 5a, when the sampling reached the 28th time (the corresponding number of characteristic wavelengths is 59), the RMSECV curve is at its lowest point, indicating that the selected group of spectral wavelengths is the best at this stage. Then, for the reflectance spectral curve, the training set divided by SPXY is used in SPA to perform second characteristic wavelength selection, and the selected characteristic wavelength is used for final modeling. The characteristic wavelengths selected are shown in Figure 5b. The characteristic wavelengths are located in the area with a significant change, which shows that the SPA can be effective. Eleven wavelengths were finally selected and their wavelengths were 826 nm, 976 nm, 985 nm, 1213 nm, 1216 nm, 1221 nm, 1826 nm, 2341 nm, 2357 nm, 2380 nm, and 2382 nm.

**Figure 6:**
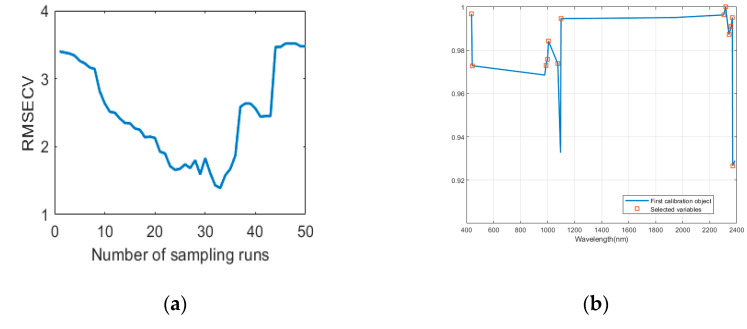
In Honghu area, the sCARS-SPA is used for characteristic wavelength selection: (**a**) the RMSE of the 5-fold cross-validation value changes with the number of sCARS algorithm sample runs; (**b**) the final characteristic wavelength and reflectance selected by SPA.

For the Honghu area, after the five-fold cross-validation, the RMSECV trends of the CR of the reflectance spectra are shown in Figure 6. As can be seen from Figure 6, when the sampling reached the 33rd time (the corresponding number of characteristic wavelengths is 17), the RMSECV curve is at its lowest point, indicating that the selected subset of spectral wavelengths is the best subset. In the same way as the Daye area, the characteristic wavelengths selected by sCARS were used by SPA to eliminate collinearity among wavelengths. Thirteen wavelengths were finally selected and the wavelengths were 437.3 nm, 441.9 nm, 987.4 nm, 999.4 nm, 1006.5 nm, 1075.3 nm, 1099.3 nm, 2303 nm, 2317.4 nm, 2341.1 nm, 2357.7 nm, 2367.1 nm and 2369.4 nm.

### 3.2. Characteristic Wavelengths

**Figure 7:**
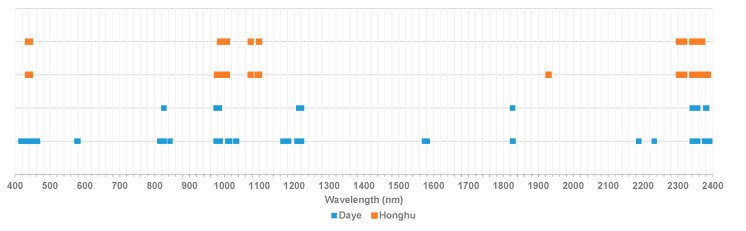
Plot of characteristic wavelengths based on sCARS and sCARS-SPA. The light blue square represents the characteristic wavelength selected by the sCARS-SPA, and the orange square represents the characteristic wavelength selected by the sCARS method.

Since the characteristic wavelengths selected by sCARS may still be redundant and have low correlation, the model may have low efficiency and accuracy. The sCARS-SPA can select the wavelength with the least redundant information as much as possible and reduce the number of characteristic wavelengths to solve the problem of information redundancy and collinearity, which improves the estimation accuracy and execution efficiency of the model. As shown in Figure 7, the characteristic wavelength of Honghu was reduced from 17 to 13, and the characteristic wavelength of Daye was reduced from 59 to 11. The sensitive wavelengths of soil As content were concentrated in 437.3–441.9 nm, 826–999.4 nm, 1006.5–1099.3 nm, 1213–1221 nm, 1826 nm, and 2303–2382 nm, which indicate that the wavelengths in these six parts were closely related to the soil As content.

### 3.3. Modeling Results

#### 3.3.1. The Result of Spectral Transformation

As a general spectral transformation, CR can effectively highlight spectral characteristics, and enhance the effect of characteristic wavelength selection algorithm. From Table 2, it can be seen that based on the CR of the reflectance spectra, the estimation accuracy of the model established by the selected characteristic wavelength based on sCARS-SPA has been significantly improved. For the Honghu area, $R_{p}^{2}$ increased from less than 0 to 0.8237 and 0.8644. For the Daye area, $R_{p}^{2}$ increased from −0.1668 and 0.2597 to 0.7002 and 0.8781, respectively. It shows that CR can better enhance the effect of sCARS-SPA and improve prediction accuracy.

#### 3.3.2. The Performance of sCARS-SPA Characteristic Wavelength Selection Algorithm

**Figure 8:**
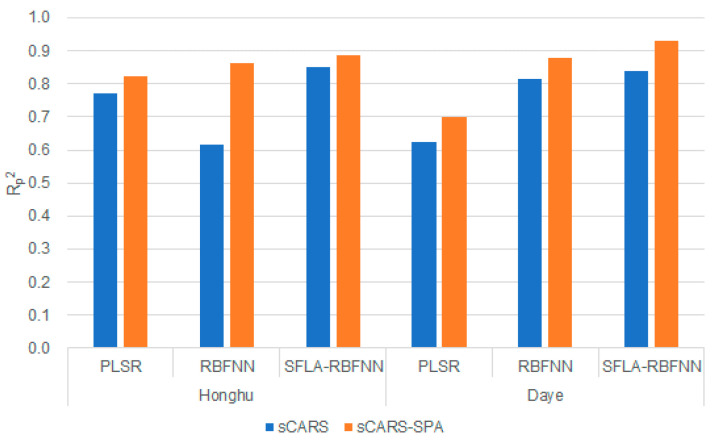
Comparison of the sCARS and sCARS-SPA in $R_{p}^{2}$.

After the CR was performed respectively, the effects of sCARS and sCARS-SPA in Honghu and Daye are compared, based on the three modeling methods of PLSR, RBFNN, and SFLA-RBFNN. Figure 8 shows the comparison of $R_{p}^{2}$. It is obvious that the sCARS-SPA model is superior to the sCARS model in terms of test accuracy. It is indicated that that the obvious collinearity in the characteristic wavelengths selected by the sCARS method is significantly eliminated after the SPA was performed.

As shown in Table 3 and Table 4, the model that obtains the highest fitting accuracy and prediction accuracy is SFLA-RBFNN. For validation set, compared to sCARS method, in the Daye area,${\;R}_{p}^{2}$ increased from 0.8403 to 0.9230, ${\mathrm{RMSE}}_{p}$ decreased from 0.3055 to 0.2161, and ${\mathrm{MAE}}_{p}$ decreased from 0.8403 to 0.1700. In the Honghu area, $R_{p}^{2}$ increased from 0.8514 to 0.8862, ${\mathrm{RMSE}}_{p}$ decreased from 0.9616 to 0.8501 and ${\mathrm{MAE}}_{p}$ decreased from 0.7810 to 0.7197. For calibration set, in the Honghu are and the Daye area, $R_{c}^{2}$ is above 0.9. And the difference between $R_{c}^{2}$ and $R_{p}^{2}$ is further smaller, avoiding the occurrence of over-fitting and under-fitting. It is obvious that sCARS-SPA improves the prediction accuracy of PLSR, RBFNN and SFLA-RBFNN, which allows these models to better predict the As content in the soil, especially the sCARS-SPA- SFLA-RBFNN model. 

#### 3.3.3. PLSR, RBFNN, and SFLA-RBNNF Model Estimation Results of Soil As Content

**Figure 9:**
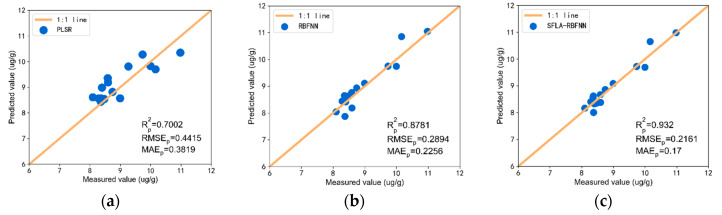
sCARS-SPA estimated results in Daye: (**a**) PLSR estimated results; (**b**) RBFNN estimated results; (**c**) SFLA-RBFNN estimated results.

In this study, the PLSR model, RBFNN model, and SFLA-RBFNN model were established and compared in different research areas. For RBFNN, the input layer is composed of the reflectivity of the characteristic wavelength selected by sCARS-SPA, and the output layer is the soil arsenic content. For SFLA, the population size is set to 100, and the maximum evolutionary generation is set to 300. As shown in Figure 9, compared with the PLSR and the RBFNN, the points of SFLA-RBF are closer to the 1:1 line and have higher prediction accuracy. In Daye, $R_{p}^{2}$ is 0.9320, ${\mathrm{RMSE}}_{p}$ is 0.2161, and ${\mathrm{MAE}}_{p}$ is 0.1700. $R_{p}^{2}$ increased by 0.0539, ${\mathrm{RMSE}}_{p}$ decreased by 0.0556, and ${\mathrm{MAE}}_{p}$ decreased by 0.0733. In addition, in areas with low As content in the soil, the prediction accuracy was more significantly improved by the SFLA method.

**Figure 10:**
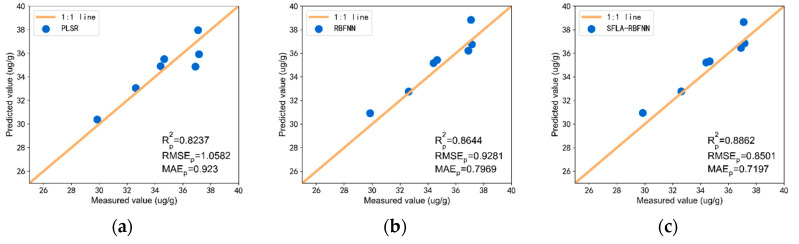
sCARS-SPA estimated results in Honghu: (**a**) PLSR estimated results; (**b**) RBFNN estimated results; (**c**) SFLA-RBFNN estimated results.

As shown in Figure 10, in the Honghu area, the SFLA-RBF model also has the highest prediction accuracy. $R_{p}^{2}$ is 0.8862, ${\mathrm{RMSE}}_{p}$ is 0.8501, and ${\mathrm{MAE}}_{p}$ is 0.7197. Compared with RBFNN, $R_{p}^{2}$ increased by 0.0218, ${\mathrm{RMSE}}_{p}$ decreased by 0.0772, and ${\mathrm{MAE}}_{p}$ decreased by 0.078. It can be seen that compared with other models, the sCARS-SPA-SFLA-RBFNN method has better robustness and accuracy and is the best model for predicting soil As content.

## 4. Discussion

Due to the presence of contaminants such as heavy metals in the soil, the spectral characteristics of the contaminated areas are slightly different from other uncontaminated areas. Hyperspectral data contain more detailed information owing to the high spectral resolution and has the good ability to identify the small differences. At the same time, a large number of bands causes the model to be complicated, reducing the prediction accuracy and efficiency. How to select characteristic bands from a large number of bands and establish a suitable and accurate model are the problems that need to be solved.

The CR-sCARS-SPA method can improve the generalization ability and accuracy of the estimation model of heavy metal content in soil while ensuring the extraction of effective feature bands. Commonly used methods based on the principle of “survival of the fittest” (such as GA [32,33], CARS [34], etc.) can select characteristics bands with strong adaptability and remove incoherent bands, but do not consider the increase in model complexity caused by the collinearity problem, which affected the prediction accuracy [35]. Before the feature selection, CR can effectively improve the accuracy of subsequent feature band selection [36,37]. It can be seen from this research that combining the SPA method with sCARS can solve the problem of data redundancy and simplify the model, thereby improving the generalization ability and prediction accuracy of the model.

Compared with other regression models, the SFLA-RBFNN model has more advantages. Soil arsenic content and spectral reflectance may often have a complex nonlinear relationship [38], so the PLSR method has poor performance in some cases. The neural network algorithm has excellent performance and efficiency, and has excellent performance in solving complex nonlinear problems [39,40,41,42], but it is prone to lack of generalization ability. Therefore, it is combined with SFLA’s excellent global search ability to optimize the initial parameters of RBFNN, so as to obtain a model with better fitting ability and prediction accuracy.

In addition, the research object of this paper is the soil samples at the sampling site, and the research scope may have certain limitations. In future research, our study will make full use of satellite and unmanned aerial vehicle remote sensing data to expand our research scope to obtain regional inversion results. Compared with the traditional spatial interpolation method based on many sampling points [43,44], the proposed algorithm combined with hyperspectral images to detect soil pollution is more economical and convenient. Moreover, based on the algorithm, other substances similar to arsenic (such as phosphorus) can also be included in the study. In addition, different forms of arsenic in soil can be estimated separately, combined with other more sophisticated detection techniques [45], which can provide the basis for comprehensive detection and protection of the soil.

## 5. Conclusions

In this study, the soil samples of Daye and Honghu were taken as the study objects. Using the two characteristic selection methods of sCARS and sCARS-SPA and the three modeling methods of PLSR, RBFNN, and SFLA-RBFNN, the soil As content was estimated based on hyperspectral data.

The research conclusions are as follows:

1. The continuous removal of the spectral reflectance of different land types can effectively enhance the effect of selecting characteristic wavelengths related to soil As content.
2. By comparing the performance of a model based on sCARS and sCARS-SPA, it is found that the model established in the wavelengths selected by sCARS-SPA has higher prediction accuracy.
3. In the two research areas, PLSR, RBFNN, and SFLA-RBFNN were used to estimate the soil As content. It was found that the SFLA-RBFNN model has highest prediction accuracy and generalization.
4. The results of experiment show that sCARS-SPA-SFLA-RBFNN model is feasible for spectral analysis of soil As content. The model not only reduces the redundancy of spectral information and solves the problem of collinearity, but also has good prediction accuracy. It provides a suitable method for the prediction of large-scale and high-precision soil As content.

## Figures and Tables

**Table 1:** Statistical characteristics of soil samples.

Study Area	Sample Size	Maximum (ug/g)	Minimum (ug/g)	Mean (ug/g)	Skewness	Kurtosis	CV (%)	SD
Honghu	27	39.21	27.08	33.05	0.05	−0.74	10.10%	3.34
Daye	62	12.84	7.04	9.28	0.59	0.38	11.96%	1.11

**Table 2:** Comparison of prediction accuracy before and after spectral pretreatment based on partial least squares regression (PLSR) and RFBNN model.

**Study Area**	**Model**	Raw Reflectance Spectra			The CR of the Reflectance Spectra		
		$RMSE_{p}$	$MAE_{p}$	$R_{p}^{2}$	$RMSE_{p}$	$MAE_{p}$	$R_{p}^{2}$
**Honghu**	PLSR	2.5715	2.286	−0.4091	1.0582	0.923	0.8237
	RBF	4.7177	4.0086	−2.6579	0.9281	0.7969	0.8644
**Daye**	PLSR	0.8235	0.6648	−0.1668	0.4415	0.3819	0.7002
	RBF	0.6886	0.5581	0.2597	0.2894	0.2256	0.8781

**Table 3:** Prediction accuracies based on sCARS.

**Study Area**	**Model**	Calibration Set			Validation Set		
		$RMSE_{c}$	$MAE_{c}$	$R_{c}^{2}$	$RMSE_{p}$	$MAE_{p}$	$R_{p}^{2}$
**Honghu**	PLSR	0.6265	0.5157	0.9688	1.1967	1.1131	0.7698
	RBFNN	0.3029	0.2280	0.9927	1.5478	1.2454	0.6149
	SFLA-RBFNN	0.5799	0.4698	0.9732	0.9616	0.7810	0.8514
**Daye**	PLSR	0.7654	0.7654	0.5426	0.5310	0.4347	0.6228
	RBFNN	0.3172	0.2586	0.9268	0.3285	0.2896	0.8154
	SFLA-RBFNN	0.3187	0.2456	0.9261	0.3055	0.2534	0.8403

**Table 4:** Prediction accuracies based on sCARS-SPA.

**Study Area**	**Model**	Calibration Set			Validation Set		
		$RMSE_{c}$	$MAE_{c}$	$R_{c}^{2}$	$RMSE_{p}$	$MAE_{p}$	$R_{p}^{2}$
**Honghu**	PLSR	0.4577	0.4577	0.9819	1.0582	0.9230	0.8237
	RBFNN	0.3182	0.2603	0.9913	0.9281	0.7969	0.8644
	SFLA-RBFNN	0.3150	0.2629	0.9914	0.8502	0.7198	0.8862
**Daye**	PLSR	0.8245	0.8245	0.5110	0.4415	0.3819	0.7002
	RBFNN	0.3543	0.2950	0.9078	0.2894	0.2256	0.8781
	SFLA-RBFNN	0.3603	0.2923	0.9046	0.2161	0.1700	0.9320

## Acronyms

CR	continuum removal
CARS	competitive adaptive reweighted sampling algorithm
sCARS	stable competitive adaptive reweighting sampling algorithm
SPA	the successive projections algorithm
sCARS-SPA	the sCARS coupled the successive projections algorithm
PLSR	partial least squares regression
RBFNN	radial basis function neural network
SFLA-RBFNN	shuffled frog leaping algorithm optimization of the RBFNN
MCS	Monte Carlo sampling
RMSE	root mean square error
RMSECV	RMSE of cross-validation
R^2^	coefficient of determination
MAE	mean absolute error

## References

[1] Khalid S., Shahid M., Niazi N.K., Rafiq M., Bakhat H.F., Imran M., Abbas T., Bibi I., Dumat C. (2017). Arsenic behaviour in soil-plant system: Biogeochemical reactions and chemical speciation influences. Enhancing Cleanup of Environmental Pollutants.

[2] Jiang Y., Zeng X., Fan X., Chao S., Zhu M., Cao H. (2015). Levels of arsenic pollution in daily foodstuffs and soils and its associated human health risk in a town in Jiangsu Province, China. Ecotoxicol. Environ. Saf..

[3] Gong Y., Qu Y., Yang S., Tao S., Shi T., Liu Q., Chen Y., Wu Y., Ma J. (2020). Status of arsenic accumulation in agricultural soils across China (1985–2016). Environ. Res..

[4] Brevik E.C., Pereg L., Steffan J.J., Burgess L.C. (2018). Soil ecosystem services and human health. Curr. Opin. Environ. Sci. Health.

[5] Chakraborty S., Weindorf D.C., Deb S., Li B., Paul S., Choudhury A., Ray D.P. (2017). Rapid assessment of regional soil arsenic pollution risk via diffuse reflectance spectroscopy. Geoderma.

[6] Zhao P., Li S., Wang E., Chen X., Deng J., Zhao Y. (2018). Tillage erosion and its effect on spatial variations of soil organic carbon in the black soil region of China. Soil Tillage Res..

[7] Phuong T.M., Lin Z., Altman R.B. (2006). Choosing SNPs using feature selection. J. Bioinf. Comput. Biol..

[8] Xu X., Ren M., Cao J., Wu Q., Liu P., Lv J. (2020). Spectroscopic diagnosis of zinc contaminated soils based on competitive adaptive reweighted sampling algorithm and an improved support vector machine. Spectrosc. Lett..

[9] Tan K., Wang H., Zhang Q., Jia X. (2018). An improved estimation model for soil heavy metal(loid) concentration retrieval in mining areas using reflectance spectroscopy. J. Soil Sediment..

[10] Wei L., Yuan Z., Zhong Y., Yang L., Hu X., Zhang Y. (2019). An Improved Gradient Boosting Regression Tree Estimation Model for Soil Heavy Metal (Arsenic) Pollution Monitoring Using Hyperspectral Remote Sensing. Appl. Sci..

[11] Fan S., Zhang B., Li J., Liu C., Huang W., Tian X. (2016). Prediction of soluble solids content of apple using the combination of spectra and textural features of hyperspectral reflectance imaging data. Postharvest Biol. Technol..

[12] Zheng K., Li Q., Wang J., Geng J., Cao P., Sui T., Wang X., Du Y. (2012). Stability competitive adaptive reweighted sampling (SCARS) and its applications to multivariate calibration of NIR spectra. Chemom. Intell. Lab. Syst..

[13] Wang S., Chen Y., Wang M., Zhao Y., Li J. (2019). SPA-Based Methods for the Quantitative Estimation of the Soil Salt Content in Saline-Alkali Land from Field Spectroscopy Data: A Case Study from the Yellow River Irrigation Regions. Remote Sens..

[14] Gholizadeh A., Žižala D., Saberioon M., Borůvka L. (2018). Soil organic carbon and texture retrieving and mapping using proximal, airborne and Sentinel-2 spectral imaging. Remote Sens. Environ..

[15] Zhao L., Hu Y., Zhou W., Liu Z., Pan Y., Shi Z., Wang L., Wang G. (2018). Estimation Methods for Soil Mercury Content Using Hyperspectral Remote Sensing. Sustainability (Basel).

[16] Yang L.I., Haidong L.I., Weisheng S. (2017). Prediction and Ecological risk assessment of heavy metals in soil based on neural network. Res. Environ. Yangtze Basin.

[17] Zhang S., Shen Q., Nie C., Huang Y., Wang J., Hu Q., Ding X., Zhou Y., Chen Y. (2019). Hyperspectral inversion of heavy metal content in reclaimed soil from a mining wasteland based on different spectral transformation and modeling methods. Spectrochim. Acta Part A Mol. Biomol. Spectrosc..

[18] Shi T., Zhang Y., Gong Y., Ma J., Wei H., Wu X., Zhao L., Hou H. (2019). Status of cadmium accumulation in agricultural soils across China (1975–2016): From temporal and spatial variations to risk assessment. Chemosphere.

[19] Li H., Liang Y., Xu Q., Cao D. (2009). Key wavelengths screening using competitive adaptive reweighted sampling method for multivariate calibration. Anal. Chim. Acta.

[20] Galvao R.K.H., Araujo M.C.U., Fragoso W.D., Silva E.C., Jose G.E., Soares S.F.C., Paiva H.M. (2008). A variable elimination method to improve the parsimony of MLR models using the successive projections algorithm. Chemom. Intell. Lab. Syst..

[21] Cai W., Li Y., Shao X. (2008). A variable selection method based on uninformative variable elimination for multivariate calibration of near-infrared spectra. Chemom. Intell. Lab. Syst..

[22] Han Q., Wu H., Cai C., Xu L., Yu R. (2008). An ensemble of Monte Carlo uninformative variable elimination for wavelength selection. Anal. Chim. Acta.

[23] Tang R., Chen X., Li C. (2018). Detection of nitrogen content in rubber leaves using near-infrared (NIR) spectroscopy with correlation-based successive projections algorithm (SPA). Appl. Spectrosc..

[24] Pullanagari R.R., Kereszturi G., Yule I. (2018). Integrating airborne hyperspectral, topographic, and soil data for estimating pasture quality using recursive feature elimination with random forest regression. Remote Sens. (Basel).

[25] Eusuff M.M., Lansey K.E. (2003). Optimization of water distribution network design using the shuffled frog leaping algorithm. J. Water Res. Plan. Manag..

[26] FAO, International Union of Soil Sciences Working Group WRB (2015). World Reference Base for Soil Resources 2014, Update 2015: International Soil Classification System for Naming Soils and Creating Legends for Soil Maps.

[27] Zhang X., Sun W., Cen Y., Zhang L., Wang N. (2019). Predicting cadmium concentration in soils using laboratory and field reflectance spectroscopy. Sci. Total Environ..

[28] Pasquini C. (2018). Near infrared spectroscopy: A mature analytical technique with new perspectives—A review. Anal. Chim. Acta.

[29] Yanfang L., Yannian L.U., Long G., Fengtao X., Yiyun C. (2016). Construction of calibration set based on the land use types in Visible and Near-Infrared (VIS-NIR) model for soil organic matter estimation. Acta Pedol. Sin..

[30] Savitzky A., Golay M.J. (1964). Smoothing and differentiation of data by simplified least squares procedures. Anal. Chem..

[31] Galvao R.K.H., Araujo M.C.U., José G.E., Pontes M.J.C., Silva E.C., Saldanha T.C.B. (2005). A method for calibration and validation subset partitioning. Talanta.

[32] Song K., Li L., Li S., Tedesco L., Hall B., Li L. (2012). Hyperspectral Remote Sensing of Total Phosphorus (TP) in Three Central Indiana Water Supply Reservoirs. Water Air Soil Pollut..

[33] Jin J., Wang Q. (2019). Evaluation of Informative Bands Used in Different PLS Regressions for Estimating Leaf Biochemical Contents from Hyperspectral Reflectance. Remote Sens..

[34] Gu X., Wang Y., Sun Q., Yang G., Zhang C. (2019). Hyperspectral inversion of soil organic matter content in cultivated land based on wavelet transform. Comput. Electron. Agric..

[35] Bai T., Chen Y., Benoît M. (2018). The Contrast of Selecting Wavelength Capability of SPA and GA for Soil EC Detecting Model. MS&E.

[36] Tan K., Ma W., Wu F., Du Q. (2019). Random forest–based estimation of heavy metal concentration in agricultural soils with hyperspectral sensor data. Environ. Monit. Assess..

[37] Liu W., Li M., Zhang M., Long S., Guo Z., Wang H., Li W., Wang D., Hu Y., Wei Y. (2020). Hyperspectral inversion of mercury in reed leaves under different levels of soil mercury contamination. Environ. Sci. Pollut. Res..

[38] Song Y., Zhao X., Su H., Li B., Hu Y., Cui X. (2018). Predicting Spatial Variations in Soil Nutrients with Hyperspectral Remote Sensing at Regional Scale. Sensors.

[39] Tian S., Wang S., Bai X., Zhou D., Luo G., Wang J., Wang M., Lu Q., Yang Y., Hu Z. (2019). Hyperspectral Prediction Model of Metal Content in Soil Based on the Genetic Ant Colony Algorithm. Sustainability (Basel).

[40] Han L., Chen R., Zhu H., Zhao Y., Liu Z., Huo H. (2020). Estimating Soil Arsenic Content with Visible and Near-Infrared Hyperspectral Reflectance. Sustainability (Basel).

[41] Wang C., Cui Y., Ma Z., Guo Y., Wang Q., Xiu Y., Xiao R., Zhang M. (2019). Simulating Spatial Variation of Soil Carbon Content in the Yellow River Delta: Comparative Analysis of Two Artificial Neural Network Models. Wetlands.

[42] Qi H., Paz-Kagan T., Karnieli A., Jin X., Li S. (2018). Evaluating calibration methods for predicting soil available nutrients using hyperspectral VNIR data. Soil Tillage Res..

[43] Martiñá-Prieto D., Cancelo-González J., Barral M.T. (2018). Arsenic mobility in As-containing soils from geogenic origin: Fractionation and leachability. J. Chem..

[44] Ma H.H., Yu T., Yang Z.F., Hou Q.Y., Zeng Q.L., Wang R. (2018). Spatial Interpolation Methods and Pollution Assessment of Heavy Metals of Soil in Typical Areas. Huan Jing Ke Xue Huanjing Kexue.

[45] Siddiqui M.F., Khan Z.A., Jeon H., Park S. (2020). SPE based soil processing and aptasensor integrated detection system for rapid on site screening of arsenic contamination in soil. Ecotoxicol. Environ. Saf..

